# Higher reinfection rate after two-stage revision arthroplasty in patients with refractory diabetes mellitus: a retrospective analysis with a minimum ten-year follow up

**DOI:** 10.1186/s12891-022-05964-9

**Published:** 2022-11-17

**Authors:** Yu-Chih Lin, Yi-Hsuan Lin, Jian-Hong Chou, Yu-Tai Lo, Chih-Hsiang Chang, Sheng-Hsun Lee, Sheng-Hsuan Lin

**Affiliations:** 1 Department of Orthopaedic Surgery, Chang Gung Memorial Hospital (CGMH), No. 5 Fu-Hsing Street, Kweishan Taoyuan, Taiwan; 2 Bone and Joint Research Center, Chang Gung Memorial Hospital (CGMH), No. 5 Fu-Hsing Street, Kweishan Taoyuan, Taiwan; 3 College of Medicine, Chang Gung University (CGU), 259 Wen-Hwa 1St Road, Kweishan Taoyuan, Taiwan; 4 Division of Endocrinology and Metabolism, Department of Internal Medicine, Chang Gung Memorial Hospital, Linkou Taoyuan, Taiwan; 5 Institute of Statistics, National Chiao Tung University, 1001 University Road, 300 Hsinchu, Taiwan

**Keywords:** Periprosthetic joint infection, Diabetes mellitus, Staged revision arthroplasty, Glycated hemoglobin

## Abstract

**Background:**

Treatment protocols for two-stage revision arthroplasty with diabetes mellitus (DM) have not yet been established. The control of glycated hemoglobin (HbA1c) in two-stage revision arthroplasty is still debated. This study aimed to clarify the importance of preoperative HbA1c levels before each stage of revision arthroplasty and to analyze the risk factors for reinfection.

**Methods:**

Five hundred eighty-eight patients suffered from first-time PJI and was treated in our institute from January 1994 to December 2010 were reviewed. The mean follow-up time was 13.8 (range, 10.2–24.8) years. Patients underwent two-stage revision arthroplasty with DM at presentation were included. The endpoint of the study was reinfection of the revision arthroplasty. Demographic, survivorship, and surgical variables were also analyzed.

**Results:**

Eighty-eight patients were identified and grouped by HbA1c level before the first stage surgery: Groups 1 and 2 had HbA1c levels < 7% and ≥ 7%, respectively. Reinfection was identified in 4.55% (2/44) and 18.18% (8/44) of the patients in Groups 1 and 2, respectively. Survivorship analysis revealed correction of the HbA1c before the final stage of revision arthroplasty as an independent factor (*p* < 0.001). The identified risks for reinfection were HbA1c levels ≥ 7% before final-stage surgery, ≥ 3 stages of revision arthroplasty, and extended-spectrum beta-lactamase (ESBL)-*Escherichia coli* PJI.

**Conclusion:**

The HbA1c level before the final stage of revision arthroplasty could affect staged revision arthroplasty outcomes. Therefore, the necessity of postponing the elective final-stage revision arthroplasty procedure for HbA1c control should be further investigated in the future.

## Background

Periprosthetic joint infection (PJI) is one of the most devastating complications following total joint arthroplasty (TJA) [1]. It has become the second main reason for revision arthroplasty, in both knee and hip arthroplasty [2]. The number of TJAs has increased recently with the evolution of arthroplasty; however, the PJI rate remains fairly constant without improvement [3]. Nonetheless, the socioeconomic costs associated with PJIs are extremely burdensome to the healthcare system. These costs are driven by comorbidities associated with repeated surgeries and the high mortalities of up to 21.12% [4]. Consequently, efforts have been made to improve treatment outcomes, with increasing research focused on PJI treatment over the last decade [5].

PJI treatment has evolved over the years [5]. The gold standard treatment for chronic PJI is two-stage revision arthroplasty. During the first stage, antibiotic-loaded bone cement (ALBC) is implanted as a spacer, following debridement with implant removal. The final-stage procedure will be scheduled at a later time after the convalescence of infection [6]. Nevertheless, the success rates for these treatment policies vary, ranging from 66 to 95% for two-stage revision arthroplasty, and are far from optimal [7]. Two-stage revision arthroplasty outcomes are well documented; however, to the best of our knowledge, the real situation and principles for patients with DM are still unclear.

The worldwide prevalence of diabetes among adults was 9.3% in 2019, and it is predicted to reach 10.2% by 2030 [8]. Meanwhile, the rate of arthroplasty in patients with DM in the USA has also been projected to increase to approximately 8% annually by 2030 [9]. Most studies have shown that inadequate perioperative glycemic control is highly associated with surgical site infection (SSI) or PJI after TJA [10]. HbA1c reflects the average plasma glucose concentration. However, controversy exists on the relationship between elevated serum HbA1c levels and SSI or PJI following TJA despite a well-known risk factor, DM [11]. Nevertheless, the American Diabetes Association (ADA) guidelines recommend an HbA1c threshold lower than 7% (53 mmol/mol) to decrease postoperative complications [12]. However, the threshold remains contentious in the orthopedics field, not only for TJA [13], but especially for two-stage revision arthroplasty. Only one study indicated that higher postoperative glucose variability during reimplantation surgery increased the risk of treatment failure [14]. Therefore, this study aimed to (1) evaluate the association between preoperative HbA1c levels at each stage of surgery and adverse outcomes following two-stage revision arthroplasty in patients with DM and (2) analyze the risk factors for reinfection of the revision arthroplasty in these patients from different viewpoints (including host, bacteria, surgery, and DM medication).

## Methods

### Database

This retrospective cohort study collected PJI patients (hip or knee) who were treated in our institute from January 1994 to December 2010. From January 1994, the database was built and these patients were followed at least ten years with institutional review board approval (IRB: 201601034B0). We used International Classification of Diseases, Ninth Revision, Clinical Modification (ICD-9-CM) code 996.66 to search these patients in hospital database. The cohort was checked by two independent orthopedic assistants. We excluded infection after fracture reduction and fixation, primary septic arthritis or synchronous PJI.

### Study sample

All PJIs following a primary hip or knee prosthesis in the database with a follow-up ≥ 10 years were investigated (*n* = 588). Only two-stage revision arthroplasty for treating first PJI were included (*n* = 421). We excluded one-stage revision arthroplasty (*n* = 45), amputation (*n* = 5), fusions (*n* = 6), and debridement, antibiotics and implant retention (DAIR)(*n* = 111) for the treatment of first PJI.

### Study design

Patients with DM experienced a PJI episode was identified in our database, and further analyzed the HbA1c level: the ADA guidelines recommend an HbA1c threshold of 7% [12] before the first stage revision arthroplasty. The patients were dichotomized into Groups 1 and 2 with HbA1c levels < 7% and ≥ 7%, respectively. Outcomes and risk factors leading to recurrent PJIs were analyzed in each group. The endpoint of the study was defined as reinfection, which was on fulfillment of the criteria of PJI mentioned later, after the staged revision surgery.

Patient demographics, comorbidities, characteristic of the previous arthroplasty, and revision arthroplasty before the PJI (if applicable), procedures to manage the PJI, and causative pathogens for PJI episodes were all recorded and analyzed. Patients who had unclear chart records, were followed for less than ten years, or did not follow the DM and PJI treatment protocols in our institute were excluded.

### Definition of terms

PJI was defined and assigned if the 2011 MSIS criteria were fulfilled [15] and was treated according to the protocols of our institute with Tsukayama’s classification [16]. If the symptoms were present for > 4 weeks, it was considered a late chronic infection. In such cases, the gold standard treatment is staged revision arthroplasty. An interval with implantation of an antibiotic-loaded bone cement spacer provides a higher success rate.

In our institute, staged revision arthroplasty proceeded according to a strict protocol following a diagnosis of late chronic infection, summarized as follows: (1) On initial presentation of PJI, if patients had systemic inflammatory response syndrome (SIRS), two sets of blood cultures were taken. (2) During the first stage of staged revision arthroplasty, we obtained multiple intraoperative cultures (three sets) with radical debridement. The prosthesis was removed and the ALBC was implanted. (3) Postoperatively, 4 weeks of systemic intravenous (IV) plus 2 weeks of oral antimicrobial agents were administered according to the culture result, on advice from an infectious disease specialist. (4) Six weeks were spent on medication “holiday.” (5) After a 3-month (in total) interim with convalescence of the PJI, joint arthrocentesis was performed. (6) Once recovery from infection was confirmed, the final staged revision arthroplasty was performed. However, if negative culture result was encountered, we used empirical antibiotics with vancomycin and ceftazidime according to our previous common bacteria report in our institute [17].

Not all PJIs resolve after two-stage revision arthroplasty; some require multiple stages. Staged revision arthroplasty was defined by the number of surgeries performed prior to final reimplantation (three-stage or four-stage revision arthroplasty). The microorganism profiles were analyzed during all PJI episodes, and polymicrobial PJI was diagnosed when more than one single species of microorganism was identified.

### *Treatment protocols for patients with HbA1c* ≥ *7%*

We controlled blood sugar levels according to the guidelines published by ADA [12]. We evaluated the patients’ underlying disease, daily schedule, eating and exercise habits, and drug adherence, and prescribed the most suitable anti-diabetic medications. We also seriously considered body weight changes and the risk of hypoglycemia. Insulin would be suggested if the HbA1c level was over 10% or there were already four different kinds of anti-diabetic drugs other than insulin. The target of sugar control varied from person to person based on the patient’s age, comorbidities, life span, and self-care function.

### Statistical analysis

Continuous variables were compared using analysis of variance if showing a normal distribution and the relationship of qualitative variables was evaluated using the chi-square or Fisher’s exact test. The risk factors contributing to treatment failure (recurrent PJI) was evaluated using univariate and multivariate logistic regression models. For all tests, *p* < 0.05 was considered significant. Processing and data analysis were performed using International Business Machines Corporation (IBM) Statistical Product and Service Solutions (SPSS), statistics for Windows, version 20.0 (IBM Corp., Armonk, N.Y., USA).

## Results

Eighty-eight patients with DM who were diagnosed with PJI were identified. These were followed retrospectively for more than ten years (range, 10.2–24.8 years) after the first-stage surgery. There was no case suffered from death during the follow-up in this cohort. These patients were further dichotomized into Groups 1 and 2 according to HbA1c level: 7% at the initial presentation of PJI (Group 1 < 7%; Group 2 ≥ 7%). There were forty-four patients in each group during the study period.

Demographic data were compared between groups (Table 1). DM medications were also analyzed between the groups (Table 2). Risk factors contributing to reinfection were an HbA1c level ≥ 7% at the final-stage surgery, ≥ 3 stages of resection arthroplasty, and the presence of ESBL-producing *Escherichia coli* (Table 3).

**Table 1:** Demographic characteristics between group 1 and group 2 at the first-stage revision arthroplasty

**Variables**	**Group 1: HbA1c < 7 (*n* = 44)**	**Group 2: HbA1c** ≥ **7 (*n* = 44)**	***p***
**Basic data**			
** Male / Female**	28 (63.6%) /16 (36.4%)	24(54.5%)/20 (45.5%)	0.759
** Age (Medium)(range)(IQR)(mean)(SD)**	73.9 (41.5,94.2)(21.13)(73.5)(14.5)	69.6 (43.6,94.9)(14.72)(68.1)(13.4)	0.258
** Body mass index(Medium)(range)(IQR)(mean)(SD)**	27.0 (18.7,40.5)(6.30)(27.6)(5.84)	26.7 (15.6,42.0)(5.53)(27.4)(6.21)	0.907
** 1st Albumin level (SD)**	3.81 (0.306)	3.63 (0.501)	0.487
** Albumin level at the last stage surgery (SD)**	3.94 (0.394)	3.78 (0.432)	0.478
** eGFR(SD)**	77.1 (37.7)	68.5 (46.7)	0.467
** 1st CRP(SD)**	92.6 (92.6)	116 (91.7)	0.311
** Last CRP(SD)**	8.31 (18.2)	23.6 (57.9)	0.177
**Functional capacity**			
** Independent (%)**	40 (90.9%)	38 (86.4%)	0.607
** Needs assistance/Dependent in ADL (%)**	2 (4.5%)	0 (0%)	
** Wheelchair (%)**	2 (4.5%)	6 (13.6%)	0.072
** Bedridden (%)**	0 (0%)	0 (0%)	
**Underlying disease**			
**Charlson comorbidity index**			
** Cancer (%)**	4 (9.1%)	2 (4.5%)	1
** Solid tumor (%)**	4 (9.1%)	4 (9.1%)	1
** Hypertension (%)**	24 (54.5%)	28 (63.6%)	0.759
** Diabetes (%)**	44 (100%)	44 (100%)	1
** Liver disease (%)**	4 (9.1%)	4 (9.1%)	1
** HCV carrier (%)**	4 (9.1%)	2 (4.5%)	1
** HBV carrier (%)**	2 (4.5%)	2 (4.5%)	1
** Alcoholism (%)**	12 (27.3%)	6 (13.6%)	0.457
** Drug user (%)**	0 (0%)	0 (0%)	1
** COPD (%)**	0 (0%)	0 (0%)	1
Renal insufficiency (%)*	10 (22.7%)	12 (27.3%)	1
** CV disease (%)**	4 (9.1%)	12 (27.3%)	0.24
** Af (%)**	4 (9.1%)	6 (13.6%)	1
** CAD (%)**	8 (18.2%)	12 (27.3%)	0.721
**Surgery related variables**			
** Operation time (resection arthroplasty) (min)(SD)**	145 (24.6)	147 (50.9)	0.69
** Operation time (revision arthroplasty) (min)(SD)**	146 (49.6)	138 (43.1)	0.391
** 1st operation blood loss(mL)(SD)**	1050 (954)	768 (862)	0.269
** 2nd operation blood loss(mL)(SD)**	1000 (796)	759 (821)	0.242
**Joint presentation**			
** Hip (%)**	32 (72.7%)	26 (59.1%)	0.525
Knee (%)	12 (27.3%)	18 (40.9%)	0.321
**Procedures**			
** 2-stage revision arthroplasty (mobile spacer)**	34 (77.3%)	40 (90.9%)	0.412
** 2-stage revision arthroplasty (static spacer)**	10 (22.7%)	4 (9.1%)	0.412
** 3-stage revision arthroplasty or more (%)**	2 (4.5%)	14 (31.8%)	0.0259^*^
** Amputation (%)**	0 (0%)	2 (4.5%)	1
**Bacteria**			
** Culture-negative**	8 (18.2%)	2 (4.5%)	0.345
** Gram positive**	28 (63.6%)	32 (72.7%)	0.746
** Gram negative**	12 (27.3%)	20 (45.5%)	0.347
** Fungus**	0 (0%)	2 (4.5%)	1
** Tuberculosis**	2 (4.5%)	2 (4.5%)	1
** Poly-microbial**	18 (40.9%)	20 (45.5%)	1
** E. Coli**	2 (4.5%)	14 (31.8%)	0.0259*
** ESBL-E. Coli**	2 (4.5%)	6 (13.6%)	0.047*
** Pseudomonas Aeruginosa**	6 (13.6%)	2 (4.5%)	0.607
** Enterococcus**	4 (9.1%)	6 (13.6%)	1
** MRSA**	4 (9.1%)	2 (4.5%)	1
**Time table**			
** Duration of IV Antibiotics (95% CI)**	33.5(-16.676–83.676)	54.6(-17.724–126.924)	0.0845
** Duration of oral antibiotics (95% CI)**	29.2(-33.52–91.92)	35.6(-34.96–106.16)	0.592
** Duration of overall antibiotics (95% CI)**	63.3(-27.84–154.44)	90.0(-26.62–206.62)	0.0935
** Appropriate empirical antibiotic within 48 h (95% CI)**	22.0 (100%)	20.0 (90.9%)	0.488

*HbA1c* glycohemoglobin, *eGFR* estimated glomerular filtration rate, *CRP* C-reactive protein, *IQR* interquartile range, *SD* standard deviation, *ADL* Activity of daily life, *HCV* hepatitis C virus, *HBV* hepatitis B virus, *COPD* chronic obstructive pulmonary disease, *CV* cardiovascular, *AF* atrial fibrillation, *CAD* coronary artery disease, *E. Coli Escherichia coli*, *ESBL* extended-spectrum beta-lactamases, *MRSA* methicillin-resistant *Staphylococcus aureus*

^*^p value < 0.05

**Table 2:** Diabetes-related characteristics between group 1 and group 2 at the first-stage revision arthroplasty

**Variables**	**Group 1: HbA1c < 7 (*n* = 44)**	**Group 2: HbA1c** ≥ **7 (*n* = 44)**	***p***
Diabetes subtype			
Type 1	0 (0%)	0 (0%)	1
Type 2	44 (100%)	44 (100%)	
Duration of Diabetes, years (SD)	9.59 (5.50)	11.5 (5.52)	0.3
Fasting glucose, mmol/l (SD)	152 (60.2)	300 (145)	< 0.001*
HbA1c (1^st^ stage), % (SD)	6.26 (0.539)	10.1 (1.68)	< 0.001*
HbA1c (last stage), % (SD)	6.32 (0.342)	6.86 (1.10)	0.116
Self-reported drug intake (N, %)	5.00 (22.7%)	9.00 (40.9%)	0.332
No glucose-lowering drugs	2 (4.5%)	4 (9.1%)	1
Anti-diabetic agent			
Metformin	18 (40.9%)	26 (59.1%)	0.366
GLP-1 receptor agonist (Liraglutide, dulaglutide, soliqua)	0 (0%)	2 (4.5%)	1
SGLT2 inhibitor (Empagliflozin, dapagliflozin, canagliflozin)	0 (0%)	2 (4.5%)	1
Sulfonylurea (Glipizide, Glimepiride, Gliclazide)	8 (18.2%)	24 (54.5%)	0.0268^*^
Glinides (Mitiglinde, Repaglinide, Nateglinide)	0 (0%)	2 (4.5%)	1
TZD (Pioglitazone, Rosiglitazone)	2 (4.5%)	2 (4.5%)	1
DPP4 inhibitor (Saxagliptin, Linagliptin, Alogliptin, Vildagliptin)	2 (4.5%)	18 (40.9%)	0.00934^*^
Acarbose	0 (0%)	12 (27.3%)	0.0211
Insulin only	0 (0%)	8 (18.2%)	0.108
Insulin combined with other OAD	4 (9.1%)	20 (45.5%)	0.0157*
ACE inhibitor or ARB	4 (9.1%)	20 (45.5%)	0.0157*
Diuretic	8 (18.2%)	10 (22.7%)	1
Calcium antagonist	16 (36.4%)	20 (45.5%)	0.759
Beta blocker	10 (22.7%)	20 (45.5%)	0.203
Other antihypertensive drug	14 (31.8%)	14 (31.8%)	1
Any antihypertensive drug	10 (22.7%)	8 (18.2%)	1
Statin	12 (27.3%)	10 (22.7%)	1
Fibrate	0 (0%)	0 (0%)	1
Antiplatelet therapy	0 (0%)	4 (9.1%)	0.488
Anti-coagulant	10 (22.7%)	12 (27.3%)	1

*HbA1c* glycohemoglobin, *SD* standard deviation, *GLP-1* Glucagon-like peptide-1, *SGLT2* sodium-glucose cotransporter 2, *TZD* thiazolidinedione, *DPP4* Dipeptidyl peptidase 4, *OAD* oral anti-diabetic drug, *ACE* angiotensin-converting enzyme, *ARB* angiotensin II receptor blockers

^*^p value < 0.05

**Table 3:** Results of multivariate logistic regression analysis of variables associated with recurrent PJI

Variables	Multivariate model results	
	**Adjusted odds ratio (95% CI)**	***p***
**1**^**st**^** Albumin**	0.11 (0.04—3.81)	0.342
**2**^**nd**^** Albumin**	0.13 (0.02—3.21)	0.231
**3-stage revision arthroplasty or more**	35.02 (1.4—50.63)	< 0.001^*^
**E. Coli**	3.67 (0.19—10.31)	0.22
**ESBL-E. Coli**	12.33 (1.16—15.55)	< 0.001^*^
**Fasting glucose, mmol/l**		
** 100 to < = 200**	1.58 (0.19—8.54)	1
** > 200**	2.16 (0.22—9.85)	0.64
** 1**^**st**^** HbA1c** ≥ 7	3.67 (0.21—3.21)	0.32
**Last stage HbA1c** ≥ 7	28.34 (1.2—40.12)	< 0.001^*^
**Sulfonylurea (Glipizide, Glimepiride, Gliclazide)**	3.21 (0.25—11.24)	0.34
**DPP4 inhibitor (Saxagliptin, Linagliptin, Alogliptin, Vildagliptin)**	2.58 (0.19—9.32)	0.32
**Insulin combined with other OAD Insulin**	5.18 (0.28—13.36)	0.12
**ACE inhibitor or ARB**	0.64 (0.09—8.95)	1

*PJI* periprosthetic joint infection, *E. Coli Escherichia coli*, *ESBL* extended-spectrum beta-lactamases, *HbA1c* glycohemoglobin, *DPP4* Dipeptidyl peptidase 4, *OAD* oral anti-diabetic drug, *ACE* angiotensin-converting enzyme, *ARB* angiotensin II receptor blockers, *CI* confidence interval

^*^p value < 0.05

**Fig. 1:**
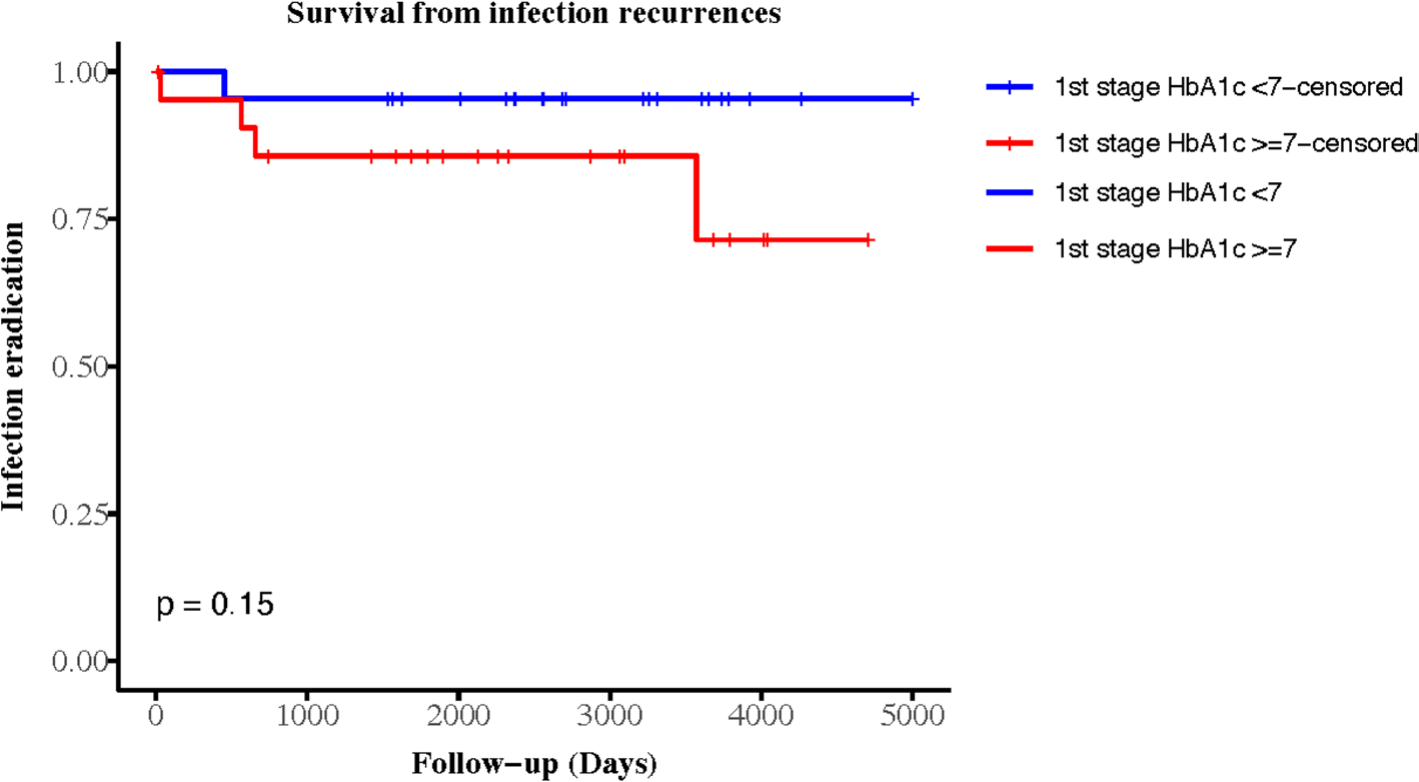
Survival curve of 2-stage revision arthroplasty free from reinfection by 1^st^ HbA1c

**Fig. 2:**
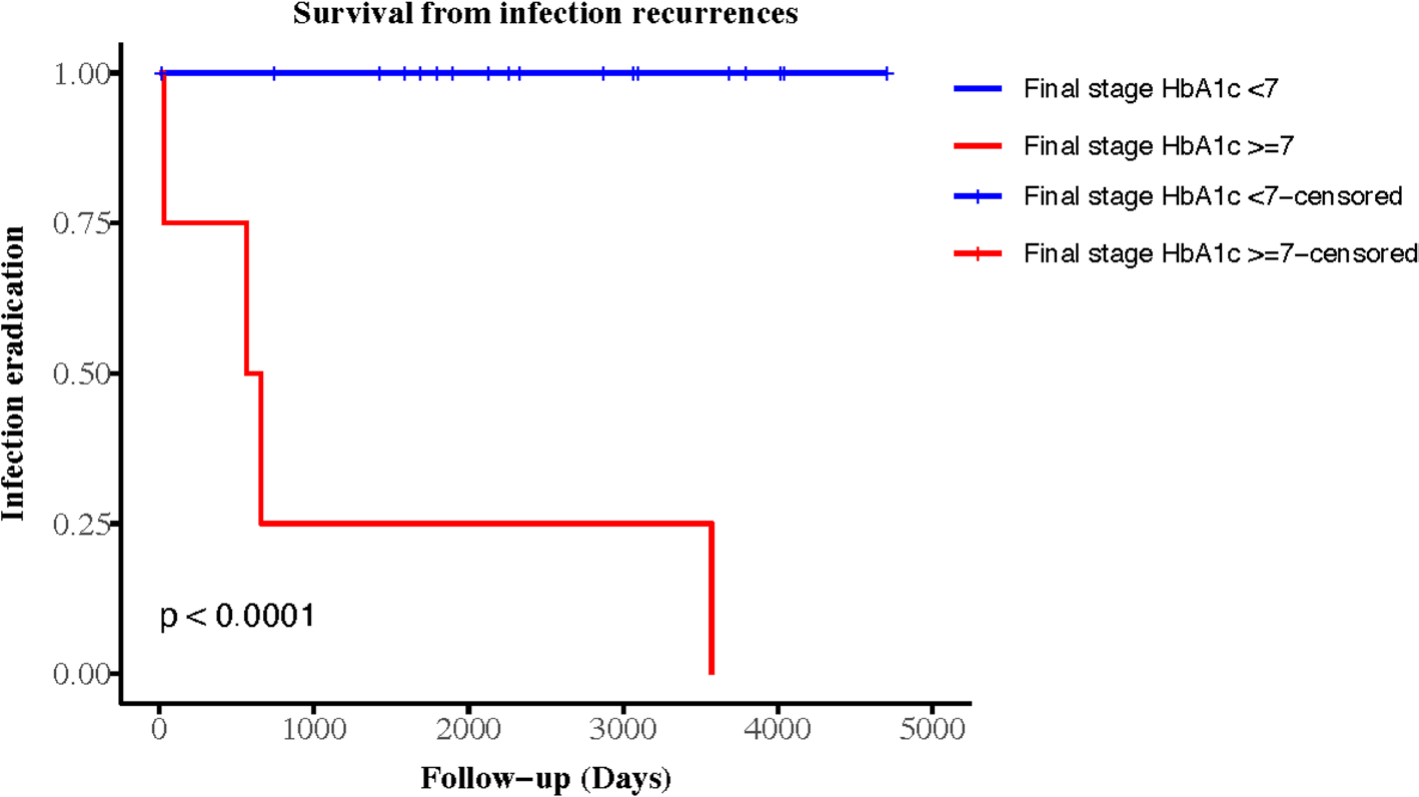
Survival curve of 2-stage revision arthroplasty free from reinfection by final stage HbA1c

Reinfection of the revision arthroplasty was identified in 4.55% (2/44) and 18.18% (8/44) of the patients in Groups 1 and 2, respectively, without significant differences in survival curves (*p* = 0.15). In Group 2, there were ten patients who followed our treatment protocol for DM and PJI and still had HbA1c levels ≥ 7% before the elective final-stage revision arthroplasty procedure. The reinfection rate in this subgroup was 80% (4/5). The Kaplan–Meier survivorship curves were analyzed by group, with a cut-off HbA1c level of 7%. Figure 1 shows the groups according to HbA1c level during the first-stage surgery for the initial PJI. Figure 2 shows groups according to HbA1c level during the final-stage procedure. The endpoint was an episode of recurrent PJI.

## Discussion

To the best of our knowledge, this is the first study to explore the role of HbA1c in staged revision arthroplasty. We found that the survivorship of staged revision arthroplasty in DM patients was favorable in those who had well controlled HbA1c levels (within 7%) before the final-stage surgery. The identified risks for reinfection after staged revision arthroplasty were HbA1c levels ≥ 7% before final-stage surgery, ≥ 3 stages of revision arthroplasty, and extended-spectrum beta-lactamase (ESBL)-*Escherichia coli* PJI.

Many studies have revealed the obscure role of HbA1c in TJA. In 2012, Richard et al. discussed DM, HbA1c, and TJA [18]. PJIs in TJA have increased in patients with DM, regardless of HbA1c level. In 2015, Hilal et al. published the results of a large database cohort study and demonstrated a significant PJI risk in patients with DM diagnoses, under DM medication, and those with perioperative hyperglycemia. However, the HbA1c level was not a risk factor for PJI. Nevertheless, other studies and guidelines have shown the importance of HbA1c; therefore, HbA1c should also be monitored (6.5–7.5%) before TJA [12]. Moreover, Jordan et al. tried in vain to identify the threshold HbA1c level in TJA [19]. In summary, the incidence of PJIs might increase; however, there is no good independent predictor of PJI after TJA.

Wang et al. reported that DM patients with increased postoperative glucose variability after two-stage revision arthroplasty had a higher chance of reinfection after revision surgery [14]. However, this association was more robust in patients without diabetes. In our cohort, patients with DM who had suffered a first PJI could be further divided into two groups based on HbA1c levels. However, an important question is whether HbA1c levels should be strictly controlled before the elective final-stage revision arthroplasty procedure. We directly compared the outcomes among DM patients with different first presentations. Unexpectedly, the division in our cohort was equal during the follow-up. The patients followed the same staged revision arthroplasty and DM treatment protocols and were attended by endocrinologists at the same institute. In summary, regardless of the patient’s HbA1c level during the first stage of revision arthroplasty, the real strategy of consequence is to control the HbA1c level to within 7% before the final stage of revision arthroplasty.

Furthermore, we investigated whether the DM medication in our series was related to reinfection rate. Table 2 summarizes the baseline DM medication during the first stage of revision arthroplasty. Although there were no significant differences in the duration of DM between the groups, the fasting glucose level before the first stage of revision arthroplasty was higher in Group 2. Moreover, endocrinologists’ prescriptions showed a higher chance of using sulfonylurea, dipeptidyl peptidase 4 (DPP4) inhibitor, acarbose, and insulin combined with an oral anti-diabetic agent to control DM at the initial presentation during the first stage of revision arthroplasty. According to the literature, the risk of PJI increased among patients receiving DM medication, but was not associated with the type of medication [20]. We cannot draw any conclusion on whether prescribed DM medication is a risk factor for reinfection; however, we have revealed that endocrinologists made great efforts to maintain HbA1c levels within the normal range in patients in Group 2. Nonetheless, there were still five patients with HbA1c levels ≥ 7% despite following our DM protocols. This is called “refractory DM.” Refractory DM could be defined as HbA1c levels ≥ 9% despite specialist care for at least six months [21]. It accounts for 15% of DM patients with some identified risk factors, such as early age of onset, number of diabetes education program visits, number of oral therapies, and insulin use [22]. This retrospective study aimed to establish whether further postponement of elective surgery on account of the HbA1c level is warranted in the future in patients under the PJI treatment protocol in our institute. A literature review revealed that surgery may help functionality and mobility, which increase activity and exercise. This indicates that postponing surgery may increase the long-term risk of DM complications [23].

In our study, undergoing ≥ 3 stages of revision arthroplasty was an independent risk factor for reinfection of the revision arthroplasty. Following the protocols in our institute, patients underwent reoperation after the first-stage surgery with repeat extensive synovectomy and debridement. Multiple surgeries may lead to contracture or scarring of the soft tissue, which might decrease susceptibility to antibiotics due to the hypovascular status [24]. A vicious cycle of resistant bacterial wound be developed with treatment failure and reinfection.

As far as this vicious cycle is concerned, ESBL-*E. coli* could be considered the second risk factor for recurrent PJI in our cohort. Tissue culture revealed significant differences only regarding ESBL-*E. coli*. Meanwhile, we also found that *E. coli* was significantly more frequently cultured in Group 2 than in Group 1. The prevalence of *E. coli* PJI is approximately 8% [25], and this resistant strain is highly related to poor outcomes in two-stage revision arthroplasty [26]. However, for DM patients with *E. coli* PJI, our study is the first to identify the relative risk. We found a higher risk of uncontrolled DM in patients with *E. coli* PJI, and ESBL-*E. coli* was an independent risk factor for reinfection after two-stage revision arthroplasty.

This study also had several limitations. First, possible missing data with selection bias might be encountered due to the retrospective case–control design. We try to minimize bias by telephone consultation of each patient followed by the same treatment protocols and rehabilitation programs. Second, we have limited cases due to the long-term follow-up. Third, we could not use the level of fructosamine in our institute yet, which could further represent the sugar levels over a 2 to 3 weeks period. This makes it a valuable marker for both screening and monitoring therapeutic interventions for TJA [27, 28]. However, the actual role in staged resection arthroplasty has not been well proven, we could investigate it in the future.

## Conclusions

HbA1c levels before the final stage of revision arthroplasty could affect staged revision arthroplasty outcomes, especially for refractory DM patients. Patients who had ≥ 3 stages of revision arthroplasty or PJI caused by ESBL-*E. coli* were at a greater risk of recurrent PJI. Therefore, the necessity of postponing the elective final-stage revision arthroplasty procedure until patients’ HbA1c levels have been controlled should be further investigated in the future.

## Data Availability

The datasets used and analysed during the current study are available from the corresponding author on reasonable request.

## Abbreviations

DM: Diabetes mellitus
HbA1c: Glycated hemoglobin
ESBL: Extended-spectrum beta-lactamase
PJI: Periprosthetic joint infection
TJA): Total joint arthroplasty
ALBC: Antibiotic-loaded bone cement
SSI: Surgical site infection
ADA: American Diabetes Association
ICD-9-CM: International Classification of Diseases, Ninth Revision, Clinical Modification
DAIR: Debridement, antibiotics and implant retention
ESR: Erythrocyte sedimentation rate
CRP: C-reactive protein
PMN%: Neutrophil percentage
SIRS: Systemic inflammatory response syndrome
IV: Intravenous
IBM: International Business Machines Corporation
SPSS: Statistical Product and Service Solutions
DPP4: Dipeptidyl peptidase 4
